# Alterations of Expression of the Serotonin 5-HT4 Receptor in Brain Disorders

**DOI:** 10.3390/ijms19113581

**Published:** 2018-11-13

**Authors:** Heike Rebholz, Eitan Friedman, Julia Castello

**Affiliations:** 1 Department of Molecular, Cellular and Biomedical Sciences, CUNY School of Medicine, New York, NY 10031, USA; Friedman@med.cuny.edu (E.F.); julia.csaval@gmail.com (J.C.); 2 Ph.D. Programs in Biochemistry and Biology, The Graduate Center, City University of New York, New York, NY 10031, USA

**Keywords:** serotonin, 5-HT 4 receptor, 5-HT4R, depression, mood disorder, expression, Alzheimer’s disease, cognition, Parkinson’s disease

## Abstract

The serotonin 4 receptor, 5-HT_4_R, represents one of seven different serotonin receptor families and is implicated in a variety of physiological functions and their pathophysiological variants, such as mood and depression or anxiety, food intake and obesity or anorexia, or memory and memory loss in Alzheimer’s disease. Its central nervous system expression pattern in the forebrain, in particular in caudate putamen, the hippocampus and to lesser extent in the cortex, predispose it for a role in executive function and reward-related actions. In rodents, regional overexpression or knockdown in the prefrontal cortex or the nucleus accumbens of 5-HT_4_R was shown to impact mood and depression-like phenotypes, food intake and hypophagia; however, whether expression changes are causally involved in the etiology of such disorders is not clear. In this context, more data are emerging, especially based on PET technology and the use of ligand tracers that demonstrate altered 5-HT_4_R expression in brain disorders in humans, confirming data stemming from post-mortem tissue and preclinical animal models. In this review, we would like to present the current knowledge of 5-HT_4_R expression in brain regions relevant to mood/depression, reward and executive function with a focus on 5-HT_4_R expression changes in brain disorders or caused by drug treatment, at both the transcript and protein levels.

## 1. Introduction

5-HT receptors are composed of 7 families (5-HT_1–7_ receptors), comprising 14 structurally and pharmacologically distinct 5-HT receptor subtypes [1]. All receptors are G-protein-coupled, with the exception of the 5-HT_3_R that belongs to the superfamily of ligand-gated ion channels. Members of all 7 receptor families are expressed in the brain: 5-HT_1_ receptors are Gα_i/0_-coupled and two receptors of this family, 5-HT_1a_R and 5-HT_1b_R, have an important function as somatodendritic autoreceptors expressed on neurons of the raphe nuclei that produce 5-HT, but they are also expressed as postsynaptic heteroreceptors in several brain areas [2]. The three members of the Gα_q/11_-coupled 5-HT_2_R family have well defined roles in the periphery such as in the vascular system and muscle contraction; however, their function in the brain is not well understood A potential link between a 5-HT_2C_R allele and vulnerability to affective disorders has been reported, and a number of antipsychotics have inverse agonist activity at 5-HT_2C_ receptors [3]. 5-HT_2C_R KO mice are highly obese and suffer from epilepsies [4]. 5-HT_2a_R mediates the hallucinatory and psychotic action of psychedelic drugs such as LSD or psylocibin [5]. Human brain Gα_i/o_-coupled 5-HT_5_R expression is localized to the cerebral cortex, hippocampus, cerebellum, and a role in mood and major depression was postulated, using pharmacological tools and knockout mice [6]. 5-HT_6_Rs are postsynaptic G_αs_-coupled receptors strongly expressed in the striatum, nucleus accumbens and cortex, and moderately in the hippocampus, amygdala, and hypothalamus. They control among others central cholinergic function [6]. The Gα_s_-coupled 5HT_7_R is mainly expressed in the limbic system, and a potential role in sleep, circadian rhythmic activity and mood has been suggested [6]. Among all 5-HT receptors, the type 3 receptor is the only ligand-gated ion channel receptor triggering rapid depolarization via the opening of non-selective cation channels. 5-HT_3_R expression in the forebrain is low, but higher levels are present in the hippocampus and amygdala [6].

5-HT_4_R was initially identified in cultured mouse colliculi cells and guinea pig brain using a functional cAMP stimulation assay [7]. In 1995, its cloning was reported [8]. Two different splicing variants, a short one, found in the striatum and a long one in the whole brain [8] were initially described, while others found the short form also present more universally in the brain [9]. 

Expression in the brain is greatest in the basal ganglia, the hippocampal formation and the cortex, as shown in human and rat brain [10,11]. 5-HT_4_R is also widely distributed in the body. Outside the CNS, it is found along the gastrointestinal tract (esophagus, ileum and colon) [12]. It is also present in the bladder, the heart and the adrenal glands. 5-HT_4_ receptors are well known for their peripheral effects on the gastrointestinal tract, and are targets in the treatment of dyspepsia, gastroesophageal reflux disease, gastroparesis or irritable bowel syndrome [13,14]. Serotonin affects heart contractility through 5-HT_4_R which is expressed in the human and pig atrium and ventricle, while, interestingly, in the rat it is only expressed in the atrium [15]. 5-HT_4_R activation leads to the contraction of heart but also to tachycardia and arrhythmia [15]. The cardiac contractile effects of 5-HT_4_R are restricted to human and pig atria and are absent from a large number of laboratory animals, such as rat, guinea pig, rabbit and frog [16]. 5-HT_4_R was also shown to be overexpressed in the cortex of the adrenal gland of a subtype of Cushing syndrome patients, a condition caused by cortisone hyper-production [17].

Compared to other serotonin receptors, the gene encoding 5-HT_4_R *(htr4)* is large and its architecture is complicated, with 38 exons spaced over 700 kb [18]. As a G-protein coupled receptor, 5-HT_4_R signals through both G protein-dependent and G protein-independent pathways. The major G protein engaged by 5-HT_4_R signaling is Gα_s_, leading to the activation of the cAMP/PKA pathway [19]. The G-protein independent non-canonical pathway activates Src and ERK kinases, leading to pERK1/2 phosphorylation [20]. 

5-HT_4_R KO mice develop normally, with no differences in body weight, metabolism, social behavior, or sleep pattern [21]. However, when stressed they exhibit reduced hypophagia [21] and re-expression of 5-HT4R in the medial prefrontal cortex rescues this phenotype [22]. In mice, 5-HT_4_R was also shown to link appetite and feeding to addiction-related behaviors since 5-HT_4_R activation in the nucleus accumbens provokes anorexia and hyperactivity, concurrently upregulating a gene induced by cocaine and amphetamine (CART) while knockdown thereof inhibits MDMA-induced hyperactivity [23].

One of the earliest functions attributed to 5-HT_4_R in rodents is its excitatory effect on acetylcholine release in the frontal cortex and the hippocampus [24,25] which was linked to its role in enhancing memory and cognition [26,27,28,29]. For example, a two-week treatment with 5-HT_4_R partial agonist RS67333 improved memory in the object recognition test in mice [30]. Olfactory associative learning was enhanced by another partial agonist (SL65.0155) in rats [31]. Other paradigms assessing social memory, autoshaping and spatial and place learning, showed a memory enhancing effect of 5-HT_4_R stimulation [29,32,33]. Conversely, receptor antagonists induced a consistent deficit in (olfactory) associative memory formation [34,35], and weakened passive avoidance memory [36]. Paralleling these behavioral changes are structural plasticity effects of potentiated learning-induced dendritic spine growth in the hippocampus in mice, an effect which is abolished by 5-HT_4_R inhibition [37].

5-HT_4_ receptors have also been found to modulate GABA and dopamine release [18,26]. Serotonin depolarizes globus pallidus neurons, increases their firing rate and alters GABA release in a 5-HT_4_R-dependent manner involving pre- and postsynaptic mechanisms [38]. In guinea pig, the 5-HT_4_R agonist BIMU-8 increased GABA release from hippocampus indirectly via cholinergic muscarinic receptors [39]. 5-HT_4_ receptors exert excitatory control on DA release in the striatum, while a receptor antagonist blocks this effect [40]. In freely moving rats, the 5-HT_4_ antagonist GR125487 significantly reduced the nigrostriatal haloperidol-induced but not basal DA outflow without affecting the mesoaccumbal DA release, indicating that 5-HT_4_R exerts facilitatory control under activated conditions [41]. This finding is also important in the context of Parkinson’s disease where the substantia nigra is selectively vulnerable to degeneration compared to the VTA, leading to a depletion in striatal dopamine. In a rat model of PD, the 5-HT_4_R agonist prucalopride selectively enhanced L-DOPA-stimulated DA release in the rat SNr and the PFC but not in the hippocampus or the striatum [42].

5-HT_4_R also impacts global serotonergic tone. 5-HT_4_R KO mice have diminished tissue levels of 5-HT and its main metabolite, 5-HIAA, increased serotonin transporter (SERT) at the protein and transcript levels, as well as decreased 5-HT_1A_R binding sites [43]. 5-HT_4_R is a component of a feedback loop projecting from the PFC to the dorsal raphe nuclei (DRN). More specifically, in mice, systemic 5-HT_4_R stimulation or overexpression of 5-HT_4_R in the mPFC increased the firing rate of DRN neurons, thus creating a positive feedback PFC-DRN loop involving 5-HT_4_R activation in cortical projections neurons, glutamate release in the DRN and enhanced DRN firing [44,45,46]. 

5-HT_4_R is a major candidate in mediating antidepressant drug action. As early as 1997, a role of 5-HT_4_R in anxiety-like behavior was described in rats [47]. More recently, this topic has received more interest, possibly due to the need to identify novel, fast acting antidepressant drugs. Indeed, it was described in rodents that subchronic (3 days) treatment with 5-HT_4_R agonist yields behavioral as well as biochemical responses in the hippocampus (CREB phosphorylation, neurogenesis) that are comparable to responses to treatment with SSRIs over 3 weeks [48], possibly through its action in the above mentioned PFC-DRN feedback loop [44,45,46]. 

These findings clearly indicate that 5-HT_4_R is a major regulator of the homeostasis of several neurotransmitter systems, implying a role in brain disorders such as Alzheimer’s, Huntington’s, Parkinson’s diseases or Major Depressive Disorder. Our review aims at summarizing the current knowledge of 5-HT_4_R expression in the brain. We also want to present knowledge on cell-type specific expression, which has not yet been studied extensively, partly due to the lack of immunohistochemistry-competent antibodies as well as resolution limits of binding experiments in brain slices with radioactive antagonists. 

## 2. Promoter Studies and Transcript Variants

Surprisingly little is known about the transcriptional regulation of the *htr4* gene across tissues. The human 5-HT_4_ receptor gene is located on chromosome 5 (5q31–q33) and contains five exons and eight alternatively spliced cassettes that code for the internal and C-terminal splice variants [16]. Human *htr4* mRNA is transcribed from a very complex gene encompassing 38 exons spanning over 700 kb [18], and multiple C-terminal isoforms are expressed in specific tissues in the CNS. To date, it is not known how 5-HT_4_ receptor expression is regulated in the brain, and so far we have only partial knowledge about the promoter, derived from human atrial tissue and placenta [16,49]. In the heart, the major transcription start site of the *htr4* gene is located at −3185 bp upstream of the first start codon [16]. In placenta, the 5′-UTR is even longer, spanning over 5100 bp upstream from the translation start site [49]. The different 5′-UTRs upstream of the translation initiation codon are interesting since they may hold an additional key to understand region and cell-type specific regulation of protein expression. 

The human promoter lacks TATA and CAAT canonical motifs, but contains several transcription factors binding sites. Transient transfection assays with human 5-HT_4_ receptor promoter-luciferase constructs identified an approx. 1.2 kb fragment of 5′-non-transcribed sequence as promoter in human cell lines but not monkey COS-7 cells [16] indicating that there is a tissue-specific expression of yet unknown transcription factors. We found in mouse brain that there is a region-specific negative transcriptional regulation of *htr4* exerted by the kinase CK2. Examination of conditional mouse knockouts of CK2 in the hippocampus, striatum and the cortex indicated an upregulation of 5-HT_4_R mRNA selectively in the cortex [46]. Furthermore, in luciferase assays, using a 4 kb element upstream of the mouse gene fused to luciferase cDNA, expression was promoted when CK2 was inhibited or knocked down in human HeLa cells but not in Hek293 or monkey COS-7 cells, again underlining the importance of tissue-specific transcription factors.

Instead of the TATA box, Maillet et al. described the presence of a sequence in the human gene (TTCACTTT) that can function as a core promoter sequence similarly to the TATA box [16]. For other species, no promoter studies were performed.

There are differential transcription initiation sites in different tissues such as human heart and placenta while for the brain no such data are yet available. While the transcription initiation start site does not affect the protein-coding region, it may alter the transcription efficiency and the expression pattern of 5-HT_4_R. It is hypothesized that such a long 5′-UTR reduces RNA translation and leads to low levels of expressed transcripts by causing premature initiation at a wrong ATG and preventing the ribosome from reaching the correct start codon [16]. 

Taken together, in particular in the human brain, there is a lack of data about the 5′-UTR, the promoter and the transcription factors that are active at the promoter for *htr4*. 

## 3. SNPs in Non-Coding Regions

In addition to the 5′-UTRs, isoforms can also vary in the 3′-UTR. These 3′-UTRs are targets for post-transcriptional regulation by non-coding RNAs such as miRNAs. Within the 3′-UTR of the 5-HT_4_R (b) and (i) isoforms from the GI tract from humans with irritable bowel syndrome (IBS), a single nucleotide polymorphism, termed 5-HT_4_R (b_2) was found to be predominantly present in a subtype of IBS patients. This isoform lacks two of the three miRNA binding sites for miR-16 family/miR-103/107 and, compared to the full length 5-HT_4_R (b) isoform, its expression yielded higher 5-HT_4_R protein levels. It was further shown that miR-16 and miR-103 are responsible for the downregulation of the transcript in vitro which is impaired in the 5-HT_4_R (b_2) mutant [50].

Another miRNA, *Let-7a*, was also postulated to have the potential to regulate 5-HT_4_R [51].

Several genome wide association studies (GWAS) and meta-analyses have associated twelve intronic SNPs in the non-coding region of human *htr4* with pulmonary function [52,53]. The same SNPs have been associated with the clinical phenotypes of airflow obstruction and COPD and asthma [53,54]. A SNP in a non-coding region could affect transcriptional regulation or generate a splicing signal. In this context, the pulmonary function of 5-HT_4_R KO mice was found to exhibit higher baseline lung resistance, confirming a role of 5-HT_4_R in airway diseases [55]. No mechanistic studies have yet been performed to understand the impact of the described SNP on transcription and splicing.

## 4. Isoforms and Alternative Splicing

In contrast to promoter-dependent transcriptional initiation sites which will still yield the same transcript but alter expression levels, splicing affects the protein sequence.

**Figure 1:**
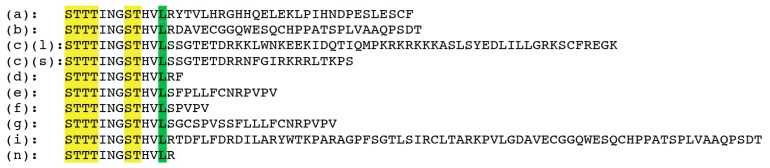
Alignment of C-termini of isoforms found in human tissue: green: leucine 358, the last amino acid common to all variants. For the c isoform, a short and a long one were described. Yellow: S/T cluster necessary for b-arrestin dependent receptor endocytosis.

Since the first publication in 1995 which described a short and a long isoform, several other isoforms were discovered: There are at least 11 human 5-HT_4_ receptor splice variants (a–i,n) [18,56,57,58,59]. All splice variants differ at the C-terminus with the exception of 5-HT_4_R (h) which is an internal splice variant with an insert in the 2nd extracellular loop [60], (Figure 1) and the (n) isoform which lacks the C-terminal exon [61].

Human 5-HT_4_ receptor isoforms (a–i and n) are highly expressed in the central nervous system [18,56,61]. Isoform (b) is the most abundant form in the CNS and periphery, and is expressed in the caudate nucleus, putamen, amygdala, pituitary gland, and small intestine. Isoform (a) is highly expressed in the amygdala, hippocampus, nucleus accumbens, and caudate nucleus and at lower levels in the small intestine, the atrium, and pituitary gland. Isoform (c) is highly expressed in the pituitary gland and small intestine and to a lesser degree in the caudate nucleus, hippocampus, and putamen. Isoform (d) is not present in the CNS but is found in the small intestine [18,61,62]. Isoform (g) seems to be highly expressed in the hypothalamus and cortex [63]. The (n) variant, which lacks the alternatively spliced C-terminal exon, is abundantly expressed in human peripheral tissues and brain regions involved in mood disorders (frontal cortex, hippocampus) [61].

Mice are currently thought to have five [64] and rats four isoforms, with the fourth, (c1) isoform expressed in the gastrointestinal tract [59,63]. In rat brain, no significant difference in expression between the long and short variants has been found by ISH [65]. The C-terminal sequences will determine the baseline activity (with the shorter isoforms being more active) or the ability to recruit binding partners such as β-arrestins and GRKs, sorting nexins or the NHERF PDZ adaptor protein [19,66,67] This will affect internalization kinetics which are different between isoforms [68]. Finally, isoforms can differ in their G protein coupling, since the 5-HT_4_R (b) isoform can couple via Gα_i_ as well as Gα_s_ [69]. 

To date, no specific isoform has been linked to a brain disorder; however, an interaction cannot be excluded since such studies have not been performed and would be very challenging. Most human studies using PET technology or radioactive labeling are based on ligands which cannot distinguish between isoforms. Quantitative RT-PCR was used to detect different isoforms and their expression in the rodent brain; however, no studies in disease models have employed this approach. The fact that mice or rats do not express the same isoforms than humans suggests that fine tuning of 5-HT_4_R signaling through a differential expression of longer or short, more active versus less active isoforms, may occur in different species.

## 5. Post-Translational Regulation

### 5.1. Phosphorylation

The amount of membrane-localized and active GPCR is a result of the ratio between receptor endocytosis and recycling. Endocytosis is initiated through (S/T) phosphorylation of GPCRs in their intracellular domains by G protein-coupled receptor kinases (GRKs) and second messenger kinases such as PKA or PKC [70]. Binding of arrestins to GRK-phosphorylated receptors results in receptor desensitization [71] and internalization [72,73,74,75,76]. 

Fourteen phosphosites in the 3rd intracellular loop and in the C-terminal tail of 5-HT_4_R that was heterologously expressed in retinal rod cells of the mouse were identified [77]; however, the identity of the kinases has not been determined. Neither has it been tested whether the phosphorylation of these sites is activity dependent.

In Hek293 cells, it was shown that GRK2 phosphorylates and desensitizes 5-HT_4_R resulting in downregulation of the cAMP/PKA pathway, while GRK5-mediated 5-HT_4_R phosphorylation resulted in reduced inhibition of ERK phosphorylation [19,78].

### 5.2. Palmitoylation

Palmitoylation is a lipid modification in which a cysteine SH group undergoes esterification with a palmitoyl group, generating an anchor to the lipid bilayer of the plasma membrane. This modification is readily reversible and, similar to phosphorylation/dephosphorylation, allows for rapid regulation of protein function, affecting GCPR endocytosis, phosphorylation, desensitization and ultimately cellular signaling. Biochemical studies in insect (Sf9) and mammalian cells (Cos7) showed that several 5-HT receptors (5-HT_1a_R, 5-HT_1b_R, 5-HT_4_R and 5-HT_7_R) are palmitoylated in their C-terminal tails. The mouse 5-HT_4_R (a) variant is palmitoylated at 3 highly conserved cysteine sites and at a C-terminal cysteine that is variant-specific. Palmitoylation near or close to protein-protein interaction motifs will affect the binding properties of the receptor, impact on constitutive activity or internalization via β-arrestin-2 [79,80].

### 5.3. Glycosylation

Only one study describes two putative N–linked glycosylation sites that conform to the consensus sequence N–X–S/T (X being any amino acid but proline) for glycosylation. These are located on the extracellular side of 5-HT_4_R, one at the N-terminus and one in the 2nd extracellular loop [77].

What is clearly missing in our understanding of all described post-translational modifications are data generated from physiologically expressed 5-HT_4_R such as in mouse brain, a comparison between brain regions and an analysis in response to drug treatment or of brain disease models. Finally, the functionality of each of these modifications should be addressed, in particular on their effect on protein stability and receptor homeostasis.

## 6. Basal Expression

### 6.1. Transcript Level

5-HT_4_R transcript expression in rodents mainly stems from in situ hybridization (ISH] experiments: In rat brain slices, ISH probes showed strong expression in the basal ganglia (caudate putamen, ventral striatum], olfactory tubercle, medial habenula and hippocampal formation while none was detected in globus pallidus and substantia nigra [65]. Similarly, human postmortem brains showed highest levels of 5-HT_4_ receptor mRNA in caudate nucleus, putamen, nucleus accumbens, and the hippocampal formation but none in globus pallidus and substantia nigra [10].

A brain-wide comprehensive appraisal of cell-specific expression is still warranted; however, some evidence has been published: Dual-label in situ hybridization for 5-HT_4_R and neuronal markers suggests expression in basal forebrain GABAergic parvalbumin synthesizing and glutamatergic cells and in glutamatergic pyramidal neurons in the medial prefrontal cortex and hippocampus of rat and guinea pig (CA1, CA3) [62,81]. 5-HT_4_R mRNA is present in 60% of rat PFC pyramidal neurons of the frontal cortex as assessed by single cell mRNA/cDNA profiling [82,83]. 

In rat hippocampal slices, the 5-HT_4_R agonist, cisapride, leads to increased hippocampal pyramidal cell activity and serotonin release, indirectly indicating that 5-HT_4_R is expressed in these cells [84]. 

### 6.2. Protein Level

Our knowledge on 5-HT_4_R protein expression stems to a large degree from radioactive ligand binding studies which for the most part has mirrored results of ISH studies. Indeed, a large number of radioligands exist that are specific to 5-HT_4_R.

High densities of [3H]-GR 113808 or [125I]-SB 207710 binding sites are present in the ventral and dorsal striatum, substantia nigra, globus pallidus and ventral pallidum, interpeduncular nucleus, islands of Calleja, and olfactory tubercle in guinea pig, mouse and rat brain, lower densities are found in the hippocampus, septal region, neocortex, amygdala and colliculi as well as habenular and several thalamic and hypothalamic nuclei [65,85,86,87]. [125I]-SB 207710 binding in the caudate putamen shows a rostrocaudal and mediolateral increasing gradient of receptor densities, paralleling that observed for mRNA localization [65]. 

Kainic acid injection into the caudate-putamen of rats to destroy GABAergic striatal projection neurons resulted in a dramatic decrease of radioactive ligand binding, suggesting that 5-HT_4_R is expressed in these neurons [11]. Similarly, 6-OHDA-lesion of dopaminergic neurons did not lead to a reduction in radioactive ligand binding but only to increased binding in the caudate putamen and globus pallidus. This allows the conclusion that 5-HT_4_R expression does not occur in DA neurons of the SN [11]. These studies were confirmed by comparing ISH and radioligand labeling data: The presence of mRNA in the rat caudate putamen and its absence in substantia nigra pars compacta and the globus pallidus suggests again that receptors found in binding studies in the caudate putamen and globus pallidus are synthesized by striatonigral and striatopallidal cells [62]. Comparison of mRNA distribution with receptor distribution as visualized with [125I]-SB 207710 further indicates that 5-HT_4_ receptors are localized somatodendritically (e.g., in caudate putamen) and on axon terminals (e.g., in substantia nigra and globus pallidus) [65,88].

Transgenic Bac-GFP mice where GFP is expressed under the 5-HT_4_R promoter are enabling a highly detailed look at protein expression in individual cells and confirm moderate to strong expression in the olfactory bulb, cerebral cortex, subicular cortex, hippocampus, striatum, globus pallidus, midbrain, pons medulla, cerebellum and weak expression in the piriform cortex, basal forebrain and the thalamus (Gensat Founder AU103). Dual immunohistochemical analysis showed expression of GFP in GABAergic spiny projection neurons but not in striatal interneurons [89]. Another transgenic mouse line where the β-galactosidase gene was knocked-in at the *htr4* gene locus shows LacZ localization in mature but not immature granule cells as suggested by staining with the neural marker, NeuN, and calbindin (mature granule cell marker) [90]. Another study confirmed that 5-HT_4_ receptors are expressed in efferent GABAergic neurons of the nucleus accumbens projecting to the lateral hypothalamus [23].

Species-specific differences of 5-HT_4_R protein expression were found between mouse/rat and guinea pig in the globus pallidus, substantia nigra and interpeduncular nucleus [87]. 

Using [3H]-prucalopride and [3H]-GR116712 or [125I]-SB 207710 in binding studies of human post-mortem brain slices, the highest densities were found in the basal ganglia (caudate nucleus, putamen, nucleus accumbens, globus pallidus, substantia nigra). Moderate to low densities were detected in the hippocampal formation and in the cortical mantle [10]. Additionally, using the labeled antagonist GR 113808, expression in the human amygdala was reported [91]. In the neocortex, the binding showed a distinct lamination pattern with high levels in superficial layers and a band displaying lower levels in deep cortical layers [92]. Membrane binding studies with [3H]-GR 113808 resulted in highest binding in the human caudate nucleus, followed by substantial densities in the lenticular nucleus, the substantia nigra, the hippocampus and frontal cortex, whereas no binding could be detected in the cerebellum [82] 

The expression data from all species studied are summarized in Table 1.

## 7. Changes in Expression in Brain Disorders and Changes Induced by Drug Treatment

In the healthy population there is a baseline difference of 5-HT_4_R protein expression between sexes. Women show lower 5-HT_4_R binding (by 13%) in the limbic system and the difference was most pronounced in the amygdala, which is highly involved in the processing and memorizing of emotions [93]. 

Studies using [3H]-GR 113808 in the rat have revealed that during development, prenatal expression is low, with the exception of the brainstem, indicating that 5-HT_4_R is largely dispensable in development. Interestingly, the synchronous appearance of 5-HT_4_ receptors and cholinergic markers validates the notion of 5-HT_4_R-mediated control over acetylcholine release [94]. 

With age, 5-HT_4_R expression goes down as older humans present lower 5-HT_4_R binding [93].

Table 2 assembles data on expression changes in disease or that are pharmacologically induced.

### 7.1. Depression and Anxiety

The understanding of the roles that 5-HT_4_ receptors play in mood disorders mainly comes from preclinical studies. Several rodent models of depression and anxiety, such as bulbectomy, glucocorticoid receptor heterozygous mice, social defeat stress or exposure to prenatal stress, all indicated changes in 5-HT_4_R expression: In mice, the experience of social defeat led to 5-HT_4_R mRNA up-regulation in the midbrain raphe nuclei and the VTA, as determined by RNA seq [105]. Similarly, restraint stress induced hypophagia and increased 5-HT_4_R mRNA levels in the medial prefrontal cortex [22]. In contrast, maternal stress led to a reduction of all mouse 5-HT_4_R variants on the mRNA level as assessed by qPCR, with the strongest difference observed for the (b) variant, while chemically induced 5-HT depletion in the embryo only affected the expression of the (b) variant in the embryonic telencephalon [106]. 

After bulbectomy, 5-HT_4_R protein binding was increased in the rat ventral hippocampus and olfactory tubercles but unchanged in the dorsal hippocampus, frontal and caudal caudate putamen. 5-HT transporter (SERT) binding was unchanged in the hippocampus and caudate putamen and slightly down in lateral septum and globus pallidus [97]. GR(+/−) mice had increased 5-HT_4_R binding in the caudal caudate putamen and the olfactory tubercles, decreased SERT binding in the frontal caudate putamen but no changes for 5-HT_4_R and SERT in the hippocampus [97]. In contrast, in the Flinders Sensitive Line, a rat model of depression, 5-HT_4_R binding was decreased in the dorsal and ventral hippocampus [100].

A 3-week long treatment regimen with the SSRI fluoxetine decreased the density of 5-HT_4 _receptor binding in the CA1 field of hippocampus as well as in several areas of the striatum in rats [101]. In contrast, 5-HT_4_R in layer 5 of the cerebral cortex was shown to be selectively upregulated after fluoxetine treatment in p11-GFP bacTRAP mice [107]. Interestingly, when 5-HT_4_R expression was quantified by qPCR on whole cortical lysate no difference in response to fluoxetine treatment was detected, while a 16-fold upregulation in the deep cortical layers was found after TRAP purification. This study clearly demonstrates that methods of purification and enrichment are necessary to achieve a resolution that is sufficient to characterize the dynamics of 5-HT_4_R expression. Given that chronic fluoxetine in mice lead to a specific upregulation in layer 5 of the cortex [107], it is clear that research into expression changes needs to be approached with techniques achieving high resolution since global expression changes might be counterweighed by cell-type and subregion-specific compensatory changes. 

Data generated in humans with [^11^C]-SB 207145 brain PET imaging suggest that 5-HT_4_R is involved in the neurobiological mechanism underlying familial risk for depression, and that lower striatal but not cortical 5-HT_4_ receptor binding is associated with an increased risk for developing major depressive disorder [108]. However, in the caudate nucleus, the relationship between 5-HT_4_R and suicide risk was inverse: Postmortem studies found increased 5-HT_4 _receptor binding in the caudate nucleus and frontal cortex of depressed suicide victims [96]. Polymorphisms of the *htr4 *gene were found to correlate with major depression and/or bipolar disorders [109]. 

A PET study showed a global reduction in cerebral 5-HT_4_R binding in healthy volunteers after a 3 week treatment with fluoxetine [110], pointing towards an inverse correlation of global 5-HT_4_R binding and synaptic serotonin levels, or an activity-induced downregulation response. 

In summary, there is strong evidence regarding the involvement of 5-HT_4_R in the etiology and expression of depression; however, different preclinical models of depression and anxiety and binding studies in humans show different responses in 5-HT_4_R expression in different brain regions that need to be further addressed.

### 7.2. Food Intake and Obesity

High levels of 5-HT_4_R are observed in obese humans [99] and in overfed rats in the caudate putamen and the nucleus accumbens shell [103]. Injection of 5-HT_4_R agonist into the nucleus accumbens reduces the drive to eat while injection of 5-HT_4_R antagonist or knockdown in the nucleus accumbens induces hyperphagia in fed mice [111]. These data suggest that changes in 5-HT_4_R expression may play a role in eating disorders. Indeed, PET studies showed a correlation between the body mass index and 5-HT_4_R protein in the nucleus accumbens, ventral pallidum, the orbitofrontal cortex and hippocampus [99]. Furthermore, the density of 5-HT_4_ receptors was found to be decreased in the temporal cortex of Alzheimer’s disease patients who also suffer from hyperphagia [112].

### 7.3. Memory and Alzheimer’s Disease

A role for 5-HT_4_R in Alzheimer’s disease has been described: The receptor was linked to APP processing and β-amyloid generation in rodent models of Alzheimer’s disease. Chronic administration of 5-HT_4_R agonists reduced β-amyloid pathology through the promotion of non-amyloidogenic cleavage of the precursor of Aβ and the consequent promotion of the neurotrophic protein, sAPPα, thereby alleviating AD pathology as well as reducing plaque load [113,114]. In a transgenic Alzheimer’s mouse model, stimulation of 5-HT_4_R exerted pro-cognitive effects, which resulted in enhanced learning through increasing acetylcholine levels [24,113,115,116]. This body of work is largely based on the use of 5-HT_4_R pharmacological tools and shows that 5-HT_4_R stimulation enhanced performance on memory tasks in rodents while receptor antagonists induced worsening of the performance on these tasks. 

During memory consolidation in a food retrieval learning paradigm, 5-HT_4_ radioligand binding showed an upregulation in olfactory lobule, caudate putamen, fundus striatum, hippocampus (CA2) and several cortical regions of young adult animals. In contrast, some but not all tested regions of older rats (hippocampal CA2 and CA3 areas, and frontal, parietal, and temporal cortex) expressed reduced 5-HT_4_ receptor density [102] pointing towards age-dependent regulation of 5-HT_4_R expression.

In humans, PET studies with [11C]-SB207145 as tracer and an episodic memory verbal learning test, resulted in an unexpected negative correlation of 5-HT_4_R and memory function in healthy young volunteers. Thus, in humans, unlike what was hypothesized based on rodent studies, fewer hippocampal 5-HT_4_Rs are representative of a better episodic memory function [98]. In newly diagnosed Alzheimer’s disease patients, 5-HT_4_R binding was positively correlated to β-amyloid burden and negatively to cognitive performance (MMSE score) suggesting that cerebral 5-HT_4_R is upregulated during preclinical stage, possibly as compensatory effect to decreased levels of interstitial 5-HT [117]. 

No preclinical studies exist to date that show changes in 5-HT_4_R expression in mouse models of Alzheimer’s disease. In humans, [^3^H]-GR 113808 labeling of post mortem brain tissue showed decreased 5-HT_4_-receptor expression in the hippocampus and prefrontal cortex in patients with Alzheimer’s disease [95]. However, another study contradicts these findings revealing no changes in 5-HT_4_R density in Alzheimer’s disease in frontal and temporal cortices [118].

Thus, to corroborate the relation between 5-HT_4_R expression and memory function in humans, in healthy and disease states, further studies are warranted.

### 7.4. Schizophrenia

Limited evidence indicates that 5-HT_4_R polymorphisms could predispose to schizophrenia [119] and attention deficit hyperactivity disorder (ADHD) [120].

### 7.5. Parkinson’s Disease

Expression of 5-HT_4_R was found to be altered in rodent models of PD. Depletion of dopamine neurons by 6-OHDA leads to increased 5-HT_4_R receptor binding in the caudal caudate-putamen and globus pallidus (+93%) [11]. In contrast, in 6-OHDA lesioned mice, 5-HT_4_R mRNA was reduced (4-fold) while L-DOPA treatment doubled the 5-HT_4_R expression in the D2-SPNs. In D1-SPNs, changes only occurred after L-DOPA treatment (2-fold) [104]. For technical reasons in this study, no comparison of the total expression levels in D1- and D2-SPNs could be made. However, these findings are very interesting since they suggest a potential role for 5-HT_4_R in L-DOPA induced dyskinesia. In post-mortem studies of PD subjects, 5-HT_4_R binding in putamen and substantia nigra was found to be unaltered [91]. The small number of patients (*N* = 6), and the non-discrimination of medication, treatment duration and disease severity does, in our opinion, not allow a conclusive statement. 

Future work involving spatially restricted deletions of 5-HT_4_ receptors or local administration of pharmacological ligands is necessary to more precisely determine the cellular and circuit-based mechanisms by which 5-HT_4_ receptors influence behavior.

## 8. Other Proteins Affecting 5-HT4R Signaling

### 8.1. SERT (5-HTT)

It is not surprising that genetic alteration of the serotonin transporter gene (5-HTT) has implications in mood disorders: For example, mice overexpressing SERT (OE) or with SERT depletion (KO) present anxiolytic-like or more anxious behaviors, respectively, when compared to WT littermates [121,122]. At the molecular level, in the homozygous SERT KO mice, the activity of the 5-HT_1A_ autoreceptor is decreased [123,124] while 5-HT_2A _receptor function is enhanced [125,126,127]. Protein levels of 5-HT_4_R are altered in the SERT KO and SERT OE mice. Precisely, autoradiography studies with [3H]-SB 207145 radioligand show increased 5-HT_4_ receptor binding in the SERT OE mice in all brain regions but the amygdala. Inversely, in the SERT KO mice, 5-HT_4_R binding is decreased in all regions studied. This is consistent with studies providing evidence that chronic treatment with SSRIs in healthy individuals decreased 5-HT4R binding as seen in PET imaging [110]. Studies in rodents replicate this result of decreased 5-HT_4_R-dependent activation of adenylate cyclase and reduced electrophysiological activity in the hippocampus [128]. In a similar fashion, mice overexpressing 5-HT_4_R in the mPFC exhibit stress-induced hypophagia and a corresponding 5-HT_4_R-dependent downregulation of SERT and 5-HT_1A_ transcripts. Oppositely, siRNA mediated knockdown of 5-HT_4_R in the mPFC induces hyperphagia [22]. 

These studies are important because they highlight that altered 5-HT concentration is most likely responsible for changes in 5-HT_4_R receptor binding as a compensatory mechanism; they also highlight the bi-directionality of this process, since exogenous alterations in 5-HT_4_R levels induce changes in 5-HT availability, negatively regulating the expression of SERT as well as serotonin receptors. 

### 8.2. Adaptor Protein p11

S100 calcium effector protein p11 (S100A10), a depression marker protein, has been identified in a yeast-based screening system as a binding partner to 5-HT_4_R, with greater affinity to 5-HT_4_R than to other serotonin receptors, such as 5-HT_1B_ and 5-HT_1D_ receptors [129]. p11 co-localizes with 5-HT_4_R in brain regions that play an important role in major depressive disorder like cingulate cortex, hippocampus, amygdala and striatum as seen by in situ hybridization and immunohistochemistry using the transgenic bac-GFP mice where GFP is expressed under the 5-HT_4_R promoter. p11 KO mice show reduced 5-HT_4_R protein in radioligand binding assays, are behaviorally less sensitive to antidepressant treatment and do not respond to 5-HT_4_R agonist. As binding partner of 5-HT_4_R and adaptor protein for many other GPCRs, p11 recruits 5-HT_4_R to the site of its action, the plasma membrane [129]. 

### 8.3. CK2

CK2 is a constitutively active and ubiquitously expressed kinase. Recently, CK2 has been identified as a negative regulator of the 5-HT_4_R [46]. Knockdown or inhibition of CK2 in vitro elevates 5-HT_4_R receptor-dependent cAMP generation and increases receptor localization at the plasma membrane in monkey COS7 cells. Interestingly, in the mouse brain, mRNA upregulation of the 5-HT_4_R is specific to the PFC. Virally-mediated focal knockdown of CK2 or overexpression of 5-HT_4_R in the mPFC generates an anti-depressed and anxiolytic-like phenotype that is similar to the phenotypes observed with CK2 knockout in the forebrain driven by Emx1-Cre or Drd1a-Cre. In addition, such conditional CK2 KO mice are more responsive to antidepressant drugs and 5-HT_4_R agonist (RS 67333) treatment [46].

### 8.4. Testosterone 

Several studies describe the relationship between sex hormones and serotonin in mood-related disorders. The prevalence of major depressive disorder is 1.7 times higher in women than in men [130]. Several studies correlate depressive episodes with hormonal changes especially in the menstrual cycle in women although the exact mechanism by which this happens is not clear [130]. In men, it has been found that plasma testosterone negatively correlates with brain 5-HT_4_R binding in humans throughout the brain [131]. Higher levels of testosterone lead to increased serotonergic signaling but whether testosterone directly regulates levels through steroid hormone receptors co-localized with 5-HT_4_R or by an indirect mechanism (e.g., increased of serotonergic tonus through other targets) to decrease expression of 5-HT_4_R needs to be further examined.

### 8.5. Nav1.7 

Nav1.7 is a voltage-gated sodium channel required for nociceptive neuronal activation. While humans lacking Nav1.7 and genetic KO mice show absence of pain, a pharmacological antagonist of this channel failed to decrease pain sensitivity, indicating that receptor signaling mediated activation of nociceptive neurons might not be the only mechanism involved in pain alleviation. For example, loss of Nav1.7 coincides with upregulation of met-enkephalin, an endogenous opioid peptide in sensory neurons, increasing opioid activity and anti-nociceptive signaling. In addition, Nav 1.7 KO mice present reduced levels of 5-HT_4_R in dorsal root ganglia [132]. Both effects, i.e., changes in enkephalin and 5-HT_4_R expression and signaling, take place in peripheral nociceptive neurons and together contribute to the analgesic effect [133].

## 9. Conclusions

It is clear that changes in 5-HT_4_R expression correlate with several disease states. In order to clarify whether these changes are also causative or involved in the etiology of disease, the expression needs to be assessed on a cellular level in preclinical models. While 5-HT_4_R overexpression in rodents, for example, through virus injection, is truly helpful in delineating the role of 5-HT_4_R in certain brain regions and cell types, these experiments have the disadvantage of introducing the gene under an exogenous promoter thus leading to non-physiological levels of expression and lacking the opportunity to study transcriptional regulation. Thus, it is preferable to study transgenic mice in which a labeled version of the receptor is expressed under its endogenous promoter such as the transgenic mouse line where the β-galactosidase gene is knocked in at the *htr4* gene locus [90], enabling unambiguous cell identification or cell-type specific purifications and quantification methods. Human PET or post mortem studies are important to verify hypotheses but may not allow the resolution needed. 

Another aspect that has to be taken into consideration is the fact that splicing variants differ between species. The factors responsible for these differences are unknown but may be important in understanding human pathologies. To bridge this knowledge gap, it would be interesting to generate, through streamlining the gene architecture by engineering/deleting of splicing sites, mice which expressing specific (human) variants only and to determine whether this will affect 5-HT_4_R-dependent phenotypes (e.g., electrophysiological properties, neurotransmitter release, receptor homeostasis, behavior and biochemical signaling cascades). Once this has been established, we will be in a better position to develop more suitable 5-HT_4_R mouse models to study human disease.

## Figures and Tables

**Table 1:** Compilation of studies of expression of the 5-HT4 receptor in human, mouse and rat brain in the basal/healthy state.

species	Tissue	Cell Type	Transcript/Protein	Method	Reference
human	caudate nucleus, putamen, nucleus accumbens, globus pallidus, substantia nigra. Lesser densities in hippocampus and cortex		protein	[3H]-R116712 and [3H]-pruclapride binding	Bonaventure et al., 2000, [10]
	caudate nucleus, putamen, nucleus accumbens, and in the hippocampal formation but not in globus pallidus and substantia nigra		mRNA	in situ hybridization	Bonaventure et al., 2000, [10]
	caudate nucleus, putamen, nucleus accumbens, globus pallidus, substantia nigra, hippocampus (CA1, subiculum), neocortex		protein	[125I-]SB 20771 binding	Varnas et al., 2003, [92],
human, calf, guinea pig	caudate nucleus, lenticular nucleus, substantia nigra, hippocampus, frontal cortex		protein	[3H]-GR 113808 binding to membrane preparations	Domenech et al., 1994, [82]
	caudate nucleus, lateral pallidum, putamen, medial pallidum, temporal cortex, hippocampus, amygdala, frontal cortex, cerebellar cortex		protein	[3H]-GR 113808 binding	Reynolds et al., 1995, [95]
rat	islands of Calleja, olfactory tubercle, ventral pallidum, fundus striati, amygdala, habenula and septo-hippocampal system. striatum, substantia nigra (lateralis), interpeduncular nucleus, superior colliculus		protein	[3H]-GR 113808 binding	Waeber et al., 1996, [94]
	caudate putamen, ventral striatum, medial habenula and hippocampus		mRNA	in situ hybridization	Vilaró et al., 1996, [65]
	prefrontal cortex	60% of pryamidal glutamatergic neurons	mRNA, protein	indirect through stimulation	Feng et al., 2001, [80]
	basal forebrain, hippocampus, cortex	GABAergic, glutamatergic and parvalbumin-containing neurons, hippocampal and cortical glutamatergic neurons	mRNA	in situ hybridization	Penas-Cazorla et al., 2015, [78]
rat, guinea pig	striatum, globus pallidaus, hippocampus, substantia nigra, olfactory tubercle		protein	[3H]-GR 113808 binding	Grossman et al., 1993, [82]
	striatum, hippocampus	striatal GABAergic projection neurons, projection from dentate granule cells to field CA3, habenulo-interpeduncular pathway, somatodendritically and axonally	mRNA, protein	[125I]-SB 207710 binding, in situ hybridization	Vilaro et al., 2005, [62]
mouse	striatum	GABAergic projection neurons but not dopaminergic neurons	protein	Kainic acid lesions and [3H]-GR 113808 binding	Compan et al., 1996, [11]
	striatum	GABAergic projection neurons	protein	immunohistochemistry	Egeland et al., 2011, [89]
	dentate gyrus	mature granule cells	protein	LacZ-IR staining	Imoto et al., 2015, [90]

**Table 2:** Compilation of studies of expression of the 5-HT4 receptor in human, mouse and rat brain in the disease state, in disease models or after drug treatment.

Species	Tissue	Condition/Treatment	Direction of Change	Transcript/Protein	Method	Reference
human	frontal cortex, caudate nucleus	suicide victims	up	protein	antagonist binding	Rosel et al., 2004, [96]
		association with bipolar disorder		SNPs	sequencing of PCR products	Ohtsuki et al.,2002, [97]
	hippocampus, frontal cortex	Alzheimer’s disease	down	protein	[3H]-GR113808 binding	Reynolds et al.,1995, [95]
	putamen	Huntington’s disease	down	protein	[3H]-GR113808 binding	Reynolds et al., 1995, [95]
	hippocampus	cognition, episodic memory, recall	negative correlation	protein	PET, [11C]-SB207145	Haahr et al., 2013, [98]
	nucleus accumbens, ventral pallidum, orbitofrontal cortex,hippocampus	body mass index, obesity	positive correlation	protein	PET, [11C]-SB207145	Haahr et al., 2012, [99]
rat	striatum, subthalamic nucleus, hippocampus	lesion of serotonergic nuclei	up in rostral dorsal, ventral striatum, substantia nigra, hippocampus	protein	[3H]-GR113808 binding	Compan et al., 1996, [11]
	striatum (caudate putamen, globus pallidus	lesion of DA neurons	up	protein	[3H]-GR113808 binding	Compan et al., 1996, [11]
	hippocampus, lateral globus pallidus	Flinders sensitive line (depression model)	down	protein	[3H]-SB207145 binding	Licht et al., 2009, [100]
	hippocampus, hypothalamus, caudate putamen, nucleus accumbens, Globus pallidus	21 days paroxetine (SSRI)	down after SSRI	protein	[3H]-SB207145 binding	Licht et al., 2009, [100]
	hippocampus, hypothalamus	4 days of 5-HT depletion	up after 5-HT depletion	protein	[3H]-SB207145 binding	Licht et al., 2009, [100]
	hippocampus (CA1), striatum	21 days of fluoxetine (SSRI)	down		[3H]-GR113808 binding	Vidal et al., 2009, [101]
	15 regions incl.hippocampus	learning: autoshaping test for food retrieval	upregulated in most regions	protein	[3H]-GR113808 binding	Manuel-Apolinar et al., 2005, [102]
	caudate putamen, nucleus accumbens	rat models of obesity	up	protein	[3H]SB207145 binding	Ratner et al., 2012, [103]
	hippocampus	maternal deprivation, unpredictable stress	down	mRNA, protein	qPCR and Western botting	Bai et al., 2014, [51]
mouse	striatum	6-OHDA lesion model of Parkinson’s disease	down in D2 MSNs	mRNA	Affymetrix GeneChip microarray	Heiman et al., 2014, [104]
	ventral hippocampus	bulbectomy	up in ventral hippocampus, down in olfactory tubercles	protein	[3H]-SB207145 binding	Licht et al., 2010, [97]
	caudal putamen	GR (+/−) mice	up	protein	[3H]-SB207145 binding	Licht et al., 2010, [97]
	midbrain raphe nuclei and VTA	social defeat	up after defeat	mRNA	RNA seq	Kudryavtseva et al., 2017, [105]
	prefrontal cortex	restraint stress	up after restraint	mRNA	qPCR	Jean et al., 2017, [22]
